# Intestinal cell migration damage induced by enteropathogenic *Escherichia coli* strains

**DOI:** 10.1590/1414-431X20187423

**Published:** 2018-07-26

**Authors:** P.A. Cavalcante, M.M.G. Prata, P.H.Q.S. Medeiros, A.V. Alves da Silva, J.S. Quetz, M.A.V. Reyes, T.S. Rodrigues, A.K.S. Santos, S.A. Ribeiro, H.N. Veras, M.D. Bona, M.S.M.G. Amaral, F.A.P. Rodrigues, I.F.N. Lima, A. Havt, A.A.M. Lima

**Affiliations:** Departamento de Fisiologia e Farmacologia, Faculdade de Medicina, Instituto de Biomedicina, Universidade Federal do Ceará, Fortaleza, CE, Brasil

**Keywords:** Enteropathogenic *Escherichia coli*, Intestinal cell migration, Type III secretion system

## Abstract

Epithelial cell migration is an essential response to enteric pathogens such as enteropathogenic *Escherichia coli* (EPEC). This study aimed to investigate the effects of EPEC infection on intestinal epithelial cell migration *in vitro*, as well as the involvement of type III secretion system (T3SS) and Rho GTPases. Crypt intestinal epithelial cells (IEC-6) were infected with EPEC strains (E2348/69, Δ*escF*, and the LDI001 strain isolated from a malnourished Brazilian child) and commensal *E. coli* HS. Wound migration and cell death assays were performed at different time-points. Transcription and expression of Rho GTPases were evaluated using real-time PCR and western blotting. Overall, EPEC E2348/69 reduced migration and increased apoptosis and necrosis levels compared to EPEC LDI001 and *E. coli* HS strains. Moreover, EPEC LDI001 impaired cell migration at a higher level than *E. coli* HS and increased necrosis after 24 hours compared to the control group. The different profiles of virulence genes between the two wild-type EPEC strains, characterized by the absence of *espL* and *nleE* genes in the LDI001, might explain the phenotypic results, playing significant roles on cell migration impairment and cell death-related events. Moreover, the type III secretion system is determinant for the inhibition of intestinal epithelial cell migration by EPEC 2348/69, as its deletion prevented the effect. Active Rac1 concentrations were increased in E2348/69 and LDI001-infected cells, while the T3SS-deficient strain did not demonstrate this activation. This study contributes with valuable insight to characterize the mechanisms involved in the impairment of intestinal cell migration induced by EPEC.

## Introduction

Enteropathogenic *Escherichia coli* (EPEC) was recently associated with higher risk of infant death in a multicenter case-control study on diarrhea in developing countries (1). In addition, the high prevalence of some strains of EPEC in both symptomatic and asymptomatic individuals from developing countries has gained significant attention (2,3).

The central mechanism of EPEC pathogenesis is a lesion characterized by attaching and effacing (A/E) and reorganization of the actin cytoskeleton of host cells, causing the destruction of intestinal microvilli (4). Furthermore, EPEC has been associated with altered intestinal arrier function, including reduction of intestinal surface area and redistribution of tight junctions (5,6). These detrimental effects occur by interactions of host cell molecules with effector proteins injected by EPEC into the epithelium through a type III secretion system (T3SS) (4). However, pathogenesis of damage due to EPEC is not well characterized (7).

Migration of crypt cells to the injured area is one of the first host responses to intestinal epithelial injury (8); members of the Rho GTPases family are required for coordinating this dynamic and complex response (9). Within this group, Cdc42 and Rac1 are involved in the formation of filopodia and lamellipodia, respectively, whereas RhoA mediates cellular contractility and formation of focal adhesions (10).

Although EPEC effector proteins, e.g., Map, EspT, and EspH, alter the function of Rho GTPases to enhance cell adhesion and promote pathogen survival (11), there is apparently no report regarding effects of EPEC on intestinal cell migration and its Rho-related alterations. Furthermore, there is a lack of information regarding major EPEC virulence factors involved in this damage. To better understand this phenomenon, we used a model of intestinal cell migration to investigate and compare the effects of two EPEC strains (the prototype E2348/69 and LDI001, isolated from a malnourished child) and a commensal *E. coli* strain (HS). We further determined whether T3SS was necessary for this effect and the role of Rho GTPases.

## Material and Methods

### Bacterial strains

Bacterial strains used in this study are listed in Table 1. The EPEC strain E2348/69 and *E. coli* strain HS were kindly provided by Dr. James Nataro, University of Virginia (USA), whereas the EPEC strains (both mutant and complemented) were graciously provided by Dr. Michael Donnenberg, University of Maryland (USA). EPEC strain LDI001 was isolated from the feces of a malnourished non-diarrheic child participating in the case-control Brazilian study from the MAL-ED network (12). Specimens were cultured on MacConkey agar plates; five colonies positive for lactose fermentation with features suggestive of E. coli were selected and characterized using biochemical tests and molecular biology assays, as previously described (13). Bacterial adherence to HEp-2 was also determined (14). Bacterial DNA was evaluated (multiplex PCRs) to detect genes encoding various virulence factors (Supplementary Table S1).

**Table 1.** Bacterial strains used in this study.

Strains	Description	References
EPEC strain E2348/69	Wild-type strain (serotype O127:H6 belonging to *E. coli* phylogroup B2), originally isolated from an outbreak of diarrhea in children, isolated in Taunton, UK	(31)
EPEC strain LDI001	Wild-type strain isolated from feces of a undernourished child without diarrhea in Fortaleza, CE, Brazil	(12)
EPEC strain UMD731	Δ*escF*; lacks functioning T3SS	(15)
Δ*escF/escF* EPEC strain	T3SS restored	(15)
*E. coli* strain HS	Nonpathogenic *E. coli*	(40)

EPEC: enteropathogenic *Escherichia coli*.

### Cell culture

The IEC-6 (CRL-1592^TM^) and HEp-2 (CCL-23) cell lines were purchased from the American Type Culture Collection (ATCC, USA). The IEC-6 cell line, derived from rat small intestinal epithelium, was used at passages 20-30. Cells were cultured in Dulbecco's modified Eagle medium (DMEM, Gibco BRL, USA) containing heat-inactivated 5% fetal bovine serum (Gibco BRL), 40 mg/mL insulin (Sigma-Aldrich, St. Louis, MO), 50 U/mL penicillin (Gibco BRL), 50 mg/mL streptomycin (Gibco BRL), and 1 mM sodium pyruvate (Gibco BRL). The HEp-2 cells (passages 9-12), derived from HeLa cell contamination and commonly used to assess bacterial adherence (14), were cultured in Minimal Essential Medium (MEM, Gibco BRL) supplemented with 10% fetal bovine serum, 100 U/mL penicillin, and 100 mg/mL streptomycin. All cells were maintained at 37°C and 90% humidity, in an atmosphere containing 5% CO_2_.

### Bacterial infections

Bacteria were cultured on MacConkey agar plates for 18-24 h and then suspended in glutamine-free DMEM at absorbances of 0.04 to 0.12 (measured at 600 nm, i.e., OD600). A portion of the culture was assessed for growth on agar plates to confirm the number of CFU per mL. The EscF-complemented strain was used as described (15). *E. coli* cultures were added to cell cultures for 3 h. Cells were then washed and glutamine-free DMEM supplemented with 200 μg/mL gentamicin was added to cultures.

### Cell migration assay

The IEC-6 cells were seeded on 12-well plates (2.5×10^5^ cells per well) and grown in DMEM. Upon reaching confluence (48 h), 5 µL of mitomycin C (final concentration 5 µg/mL; Roche, USA) was added to each well and cells were incubated at 37°C for 15-20 min. Then, cells were washed, and the monolayer was cut (sterile blade) from the center of the well to the periphery. Wells were washed again, and cells were infected according to the protocol described above. Cells were washed once with PBS, and at 2, 6, 12, and 24 h after treatment with gentamicin were photographed using an inverted microscope (Nikon E400, Japan) at a magnification of 10×. The number of migrating cells was counted in an area of 0.1 mm^2^ overlaying the center of the cut, as previously described (16). Images were analyzed using ImageJ Pro Plus software, version 5.0 (Media Cybernetics, USA).

### Flow cytometric analysis of apoptotic and necrotic cell death

A RC-Anxf-T100 Apoptosis Assay Kit (Exbio, CZ) was used to quantify apoptotic and necrotic cells. Cells were collected 2, 6, 12, and 24 h after infection. Cells were trypsinized, centrifuged, and washed before incubation with annexin V and propidium iodide (PI) to detect apoptotic and necrotic cells, respectively. Samples were processed using a FACS Calibur flow cytometer (Becton Dickinson, USA), and data analyzed with CellQuest¯ software (Becton Dickinson).

### Quantitative PCR analysis

Total RNA was isolated using the RNeasy Plus Mini Kit (Qiagen, Inc., USA) from uninfected IEC-6 cells or cells infected for 3 h with 10^6^ CFU per mL of each EPEC strain. The iScript cDNA Synthesis Kit™ (Bio-Rad, USA) was used to synthesize cDNAs according to the manufacturer's instructions. Transcriptions of *RhoA, Rac1*, and *Cdc42* were used to investigate mechanisms of crypt cell migration (17) using the IQ5 Real-Time PCR Detection System (Bio-Rad). The reference gene *ywhaz* encoding phospholipase A_2_ was used for these experiments (18). All primers and conditions for qPCR are shown in Table 2. The qPCR reactions were performed in reaction mixtures containing 10 µL of iQ Supermix (Bio-Rad) in a standard amplification buffer containing optimal concentrations of DNA polymerase, dNTPs, buffer, and saline, plus 2 µL of each primer pair (0.2 mM), 1 µL of cDNA, and nuclease-free water (final volume of 20 µL). To ensure amplification specificity, each reaction was performed by increasing the temperature by 0.5°C at 15 s intervals, starting from the annealing temperature of the primers and ending at 95°C (71 cycles). Data were analyzed with the ΔΔCt method (19).

**Table 2.** Description of genes, GenBank accession numbers, RT-qPCR primers, and PCR conditions.

Gene	GenBank accession number	Primer sequences (5′-3′)	PCR Conditions	References
*rhoA*	NM_057132.3	TGGTGATGGAGCTTGTGGTAAG AACATCAGTGTCTGGGTAGGAG	20s-95°C 20s-58.5°C 45s-72°C	(17)
*rac1*	NM_134366.1	CAGCTGGACAGGAAGATTATGAC CCACTAGGATGATGGGAGTATTG	20s-95°C 20s-61°C 45s-72°C	(17)
*cdc42*	NM_171994.	GCTTGTCGGGACCCAAATTG ACACCTGCGGCTCTTCTTCG	20s-95°C 20s-61°C 45s-72°C	(17)
*ywhaz*	NM_013011.3	GCTACTTGGCTGAGGTTGCT TGCTGTGACTGGTCCACAAT	20s-95°C 20s-60°C 45s-72°C	(18)

In all qPCR reactions, the initial denaturation step was the same for all primers (95°C for 3 min).

### Western blot analysis and active Rac1

Western blotting was performed as described (15), with some modifications. Briefly, IEC-6 cells were grown on plates with 12 wells for 48 h and then infected with EPEC strains for 3 h. Cells were scraped in RIPA lysis buffer supplemented with protease inhibitor (Sigma) on ice. The suspension was centrifuged (10 min, 4°C, 180 *g*) and the supernatant collected. Protein concentrations were measured using PierceTM BCA protein assay kit (Thermo Scientific, USA). Then, proteins were separated by an SDS-polyacrylamide and transferred to polyvinylidene difluoride (PVDF) membranes by electrophoresis. The PVDF membranes were blocked with 5% bovine serum albumin for 1 h and incubated overnight with the following primary antibodies: anti-Rac1 mouse monoclonal IgG (1:250) and anti-GADPH (glyceraldehyde-3-phosphate dehydrogenase) rabbit polyclonal IgG (1:500; Santa Cruz Biotechnology, USA). Membranes were washed in Tris-buffered saline with Tween and then incubated for 1.5 h with secondary antibodies (diluted 1:500). Chemiluminescent detection using Clarity Western ECL Substrate (Bio-Rad) was performed, and bands were captured using the ChemiDoc system (Bio-Rad). Densitometric quantification of bands was done with Image J software, version 1.6.0 (National Institutes of Health, USA). To measure Rac1 activation, a pull-down assay, the rhotekin binding domain affinity precipitation for Rac1-GTP, was used according to the manufacturer's protocol (Cytoskeleton, USA) and SDS-PAGE was performed as described above.

### Statistical analysis

Experiments were performed on three days, and data are reported as means±SE. Differences between groups were compared with one-way ANOVA (followed by Bonferroni's post-test) or an unpaired Student's *t*-test. All analyses were performed using GraphPad Prism, version 5.0 (GraphPad, USA). The confidence interval was 95%, and differences were considered significant when P<0.05.

## Results

### EPEC strain LDI001 displayed localized adherence and harbored several EPEC virulence genes

Before the use of experimental infections, virulence of EPEC strains was characterized. Both EPEC strains (E2348/69 and LDI00) displayed localized adherence patterns on HEp-2 cell lines, whereas the commensal strain (HS) had no particular pattern of adhesion (data not shown). Furthermore, both EPEC strains equally harbored most virulence genes (*eae*, *bfpA*, *espB*, *espD*, *tir*, *nleB*, *map*, *espJ*, *espL*, *espC*, *espZ*, *espH*, *ler*, *espG*, *nleE*, *nleF*, *cesT*, *espP*, *nleD*, and *nleC*), except for *espL* and *nleE* genes, which were present only in the prototype strain.

### EPEC strains impaired migration of IEC-6 cells

**Figure 1.**
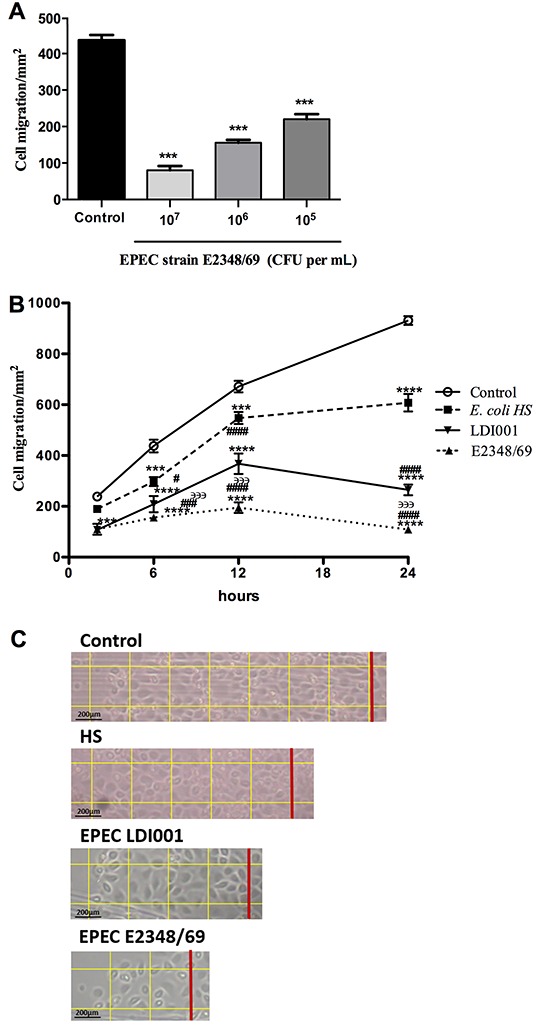
Effects of bacteria on IEC-6 cell migration. *A*, Concentration analysis of enteropathogenic *Escherichia coli* (EPEC) strain E2348/69 with 10^5^, 10^6^, and 10^7^ cell forming units (CFU) per mL on cell migration at 6 h to determine optimum inoculum. *B*, Time-course analysis of damage on intestinal epithelial cells migration induced by EPEC strain E2348/69 and LDI001, and commensal *E. coli* strain HS (inoculum, 10^6^ CFU per mL). Data are reported as mean±SE of three independent experiments (n=8). *C*, Intestinal cell migration after 24 h. The red line represents the initial cut and each square has an area of 0.1 mm^2^. Magnification 10×, bar: 200 μm. ***P<0.001 and ****P<0.0001 *vs* uninfected control; ^#^P<0.05, ^###^P<0.001, and ^####^P<0.0001 *vs E. coli* strain HS; ^϶϶϶^P<0.001 *vs* EPEC strain LDI001 (ANOVA followed by Bonferroni's post-test).

To determine optimal inoculum size for migration analysis, infections were done using MOI (multiplicity of infection) 1, 10, and 100 of EPEC E2348/69. Reductions in migrated cells after 6 h was dependent on the size of the inoculum; 50% of the maximum reduction occurred using an inoculum of 10^6^ CFU per mL, which was chosen for subsequent tests (MOI=10:1) (Figure 1A). Dynamics of migrating cells infected with either EPEC strain and the commensal *E. coli* strain were evaluated after 2, 6, 12, and 24 h. Migration of IEC-6 cells was significantly reduced (compared to a control group) by both EPEC strains at all time-points. In addition, after 6 h, cell migration was reduced by strain E2348/69 compared to LDI001 (P<0.0001; Figure 1B). Furthermore, *E. coli* strain HS decreased wound migration after 6 h compared to the control group (P<0.0001; Figure 1B). Both EPEC strains inhibited cell migration after 6 h compared to *E. coli* strain HS (P<0.05; Figure 1B). Intestinal cell migration rates from EPEC (E2348/69 and LDI001) strains and *E. coli* HS infection are observed by representative 24-h microphotographs, as shown in Figure 1C.

### Inhibition of intestinal epithelial cell migration by EPEC strains may involve apoptosis and necrosis

**Figure 2.**
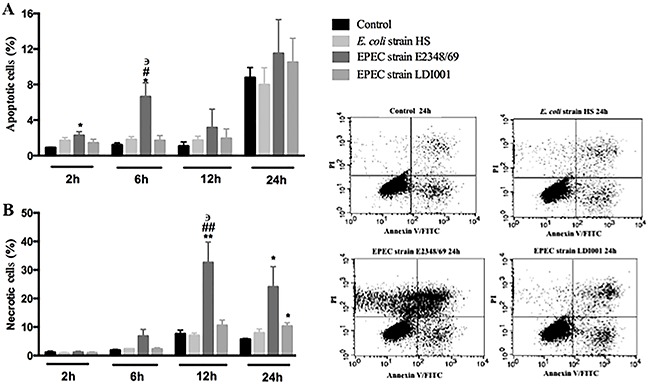
Kinetic analysis of apoptosis (*A*) and necrosis (*B*) rates on intestinal epithelial IEC-6 cell induced by enteropathogenic *Escherichia coli* (EPEC) strains E2348/69 and LDI001 and commensal *E. coli* strain HS (inocula, 10^6^ CFU per mL). Data are reported as mean±SE of three independent experiments. *P<0.05 and **P<0.01 *vs* uninfected control; ^#^P<0.05 and ^##^P<0.01 *vs E. coli* strain HS; ^϶^P<0.05 *vs* EPEC strain LDI001 (ANOVA followed by Bonferroni's post-test).

There was a greater percentage of apoptotic cells 2 h (2.27%) and 6 h (6.63%) after infection with EPEC strain E2348/69 compared to uninfected cells (0.91%, P=0.0338 and 1.21%, P=0.0258, respectively). Additionally, EPEC strain E2348/69 (6.63%) significantly increased cell apoptotic rate relative to *E. coli* strain HS (1.81%, P=0.038) and EPEC strain LDI001 (1.70%, P=0.0403) at 6 h. All groups similarly increased apoptotic cells, with s rates averaging ∼10% at 24 h (Figure 2A). Moreover, the percentage of necrotic cells was greater in cells infected with EPEC strain E2348/69 (32.61%; 24.07%) compared to the uninfected group at 12 and 24 h (7.61%, P=0.0263 and 5.71%, P= 0.0292). Furthermore, the percentage of necrotic cells at 12 h after infection with EPEC strain E2348/69 was increased significantly compared to other infected groups. Moreover, EPEC strain LDI001 (10.33%) had a significantly higher necrotic cell rate than a control group (5.71%, P=0.0184) at 24 h (Figure 2B), although there was no significant difference in necrosis between the *E. coli* strain HS and a control group.

### EPEC-induced decrease in intestinal cell migration was T3SS-dependent

**Figure 3.**
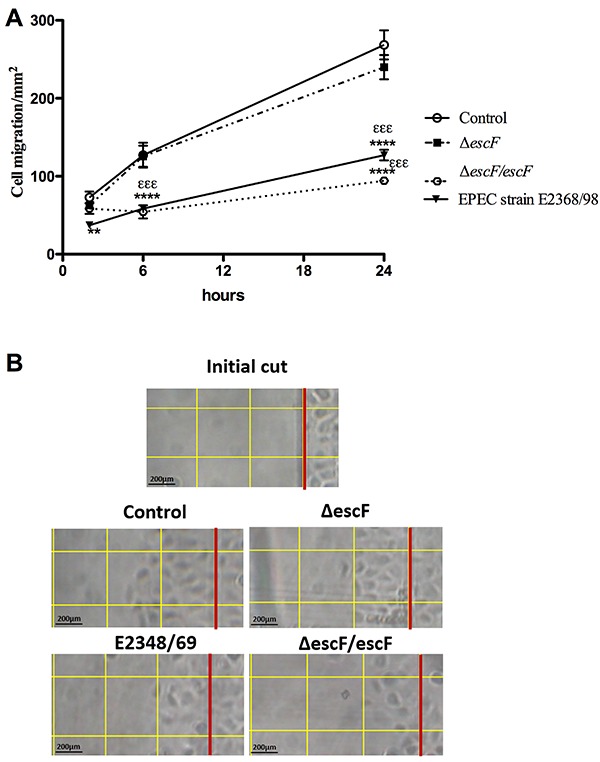
Effects of enteropathogenic *Escherichia coli* (EPEC) strain E2348/69, EscF-complemented (Δ*escF*/*escF*), and type III secretion system (T3SS)-deletion mutant (Δ*escF*) infection on migration of IEC-6 cells *A*. Data are reported as mean±SE of three independent experiments (n=7). **P<0.01 and ****P<0.001 *vs* uninfected control; ^εεε^P<0.001 *vs* ΔescF EPEC strain (ANOVA followed by Bonferroni's post-test). *B*, Intestinal cell migration after 6 h. The red line represents the initial cut and each square has an area of 0.1 mm^2^. Magnification 10×, bar: 200 μm.

To determine whether the reduction in intestinal migration was mediated by T3SS, a Δ*escF* EPEC strain, which lacks functioning T3SS, was used. At 2 h, the number of migrating cells infected with EPEC strain E2348/69 (37.14±3.59) was decreased compared to a control group (72.85±7.46, P=0.0013). Furthermore, at 6 and 24 h, E2348/69 and escF complemented EPEC strains significantly reduced cell migration compared to ΔescF EPEC (P<0.001) and control groups (P<0.0001). It was noteworthy that the T3SS-deficient (ΔescF) EPEC strain did not impair cell migration at any time-point (Figure 3A). Representative microphotographs of the intestinal migration cells infected by Δ*escF* and EscF-complemented strain EPEC at 6 h are shown in Figure 3B.

### Regulation of Rho GTPases in intestinal cell migration after EPEC infection

**Figure 4.**
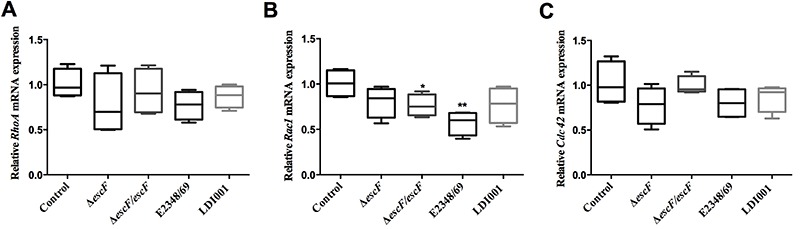
Expression of the transcription of small Rho GTPases *RhoA* (*A*), *Rac1* (*B*), and *Cdc42* (*C*) by IEC-6 cells after infection with enteropathogenic *Escherichia coli* (EPEC) strain E2348/69, EscF-complemented (Δ*escF*/*escF*) and T3SS-deletion mutant (ΔescF). Results were determined by quantitative RT-PCR and normalized to *ywhaz* (housekeeping gene) and were performed by the ^ΔΔ^CT method. Data are reported as means±SE of three independent experiments. *P<0.05 and **P<0.01 *vs* uninfected control (ANOVA followed by Bonferroni's post-test).

**Figure 5.**
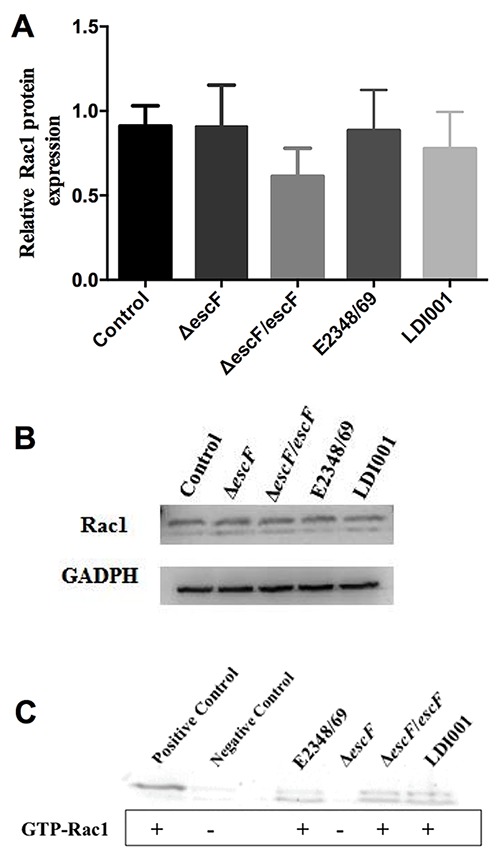
Enteropathogenic *Escherichia coli* (EPEC) strains E2348/69, EscF-complemented (Δ*escF/escF*) and T3SS-deletion mutant (Δ*escF*) infection did not affect protein expression of Rac1 in IEC-6 cells (western blot analysis) (*A* and *B*). EPEC strains, except the type III secretion system (T3SS)-deficient strain, promoted Rac1 activation (*C*). Data are reported as means±SE of three independent experiments.

After 3 h of EPEC infection, *Rac 1* mRNA levels were decreased in cells infected with EPEC E2348/69 (-1.76-fold, P=0.005) and *escF* complemented strains (-1.31-fold, P=0.0457) compared to uninfected cells (Figure 4B). However, there was no significant difference in *RhoA* and *Cdc42* transcription between infected and uninfected IEC-6 cells (Figure 4A and C). Protein Rac 1 was not altered in any group (Figure 5A and B). However, all pathogenic strains had active Rac1, although this was absent in T3SS-deficient strains (Figure 5C).

## Discussion

In this study, we aimed to investigate the intestinal cell migration impairment caused by two different EPEC strains. We also assessed the involvement of T3SS and Rho GTPases in the process. Overall, we demonstrated that EPEC E2348/69 and LDI001strains inhibited small intestinal epithelial cells migration *in vitro*. Additionally, necrosis and apoptosis apparently affected this phenomenon. Differential effects of EPEC strains on epithelial injury suggested *espL* and *nleE* virulence genes as having potential roles in causing increased damage. Furthermore, T3SS deletion in EPEC E2348/49 did not alter cell migration, and expression of Rho GTPases was not involved.

This was apparently the first study to clarify the dynamics of EPEC effects on intestinal cell migration. Although some studies reported a decrease in migration of stomach cells infected with EPEC (20,21), mechanisms were not investigated. Our group described that infection by enteroaggregative *E. coli* (EAEC) decreased the number of migrating intestinal cells (22), whereas, in the present results, we determined that EPEC strains significantly reduced the number of migrating cells and that bacterial strains differentially inhibited migration. The most considerable reduction in cell migration was due to infection with EPEC strain E2348/69, compared to EPEC LDI001 and *E. coli* HS strains. In addition, we included a commensal strain to ensure that this was a specific effect of the presence of bacteria or secreted bacterial molecules. Interestingly, *E. coli* strain HS also inhibited migration of intestinal cells; however, this effect was significantly less pronounced compared to pathogenic strains. Bacterial lipopolysaccharide significantly inhibited migration of IEC-6 cells across a wound, both in an animal model and *in vitro,* and this may explain the *E. coli* HS findings (23).

We determined whether cell death affected migration of intestinal cells. Initially, cell death caused by EPEC strains E2348/69 caused a slight increase in the percentage of apoptotic cells at early time-points, whereas strain LDI001 did not induce apoptosis, suggesting that other bacterial factors are necessary to decrease cell migration. In contrast, high (E2348/69) and moderate (LDI001) rates of necrosis at the end of the experiment may have had a greater impact on reductions in wound migration. In an *in vitro* study using *Clostridium difficile* toxin A, inhibitory effects on cell migration were associated with cell death (24). Our present results agree with those of Abul-Milh et al. (25) who reported increased necrosis in cells infected with EPEC strain E2348/69. Moreover, some virulence factors, e.g., BFP, Map, and EspH, were shown to induce cell death (26), and both EPEC strains in the present study had genes for these effectors. Thus, these genes could be responsible for cell death induction by the strains studied, however specific mutant strains should be constructed to prove such hypothesis.

Interestingly, we identified varying degrees of impairment in cell migration and cell death caused by the two wild-type EPEC strains. EPEC strain E2348/69 induced greater epithelial injury than EPEC strain LDI001. The different profiles of virulence-related genes between these strains, notably the absence of *espL* and *nleE* genes in the EPEC strain LDI001 (whereas EPEC E2348/69 harbors all of them), suggests a vital role of these virulence factors on the EPEC-induced epithelial injury, and the use of specific mutant strains could confirm this finding. The EspL effector is encoded by two genes, *espL1* (pseudogene) and *espL2* (both targeted by primers used in this study). Of these, *espL2* is very similar to EHEC *espL2,* which interacts with annexin-2, leading to condensation of actin fibers and formation of a pseudopod-like structure (27). NleE, a non-LEE effector protein, is also encoded by the genes *nleE1* and *nleE2* (both targeted by primers used in this study) and seems to modulate the immune response through inhibition of NF-kB by a T3SS-dependent mechanism (11,28). Additionally, it induces polymorphonuclear leukocyte transepithelial migration *in vitro* (29). In support of our data, in a broad investigation of EPEC virulence factors by oligonucleotide array in strains isolated from children from a case-control study of diarrhea in Norway, genes from a pathogenicity island, including *nleE* and *espL*, were significantly more common in isolates from children with diarrhea (30). It is noteworthy that the EPEC strain E2348/69 was initially isolated from an acute diarrhea case (31), whereas LDI001 was isolated from a malnourished child without diarrhea. Although further genomic investigations are required to distinguish genetics of these EPEC strains, this study demonstrated a clear evidence of these differences. Additionally, clear outcomes from each strain were consistent with different effects on intestinal epithelial cells. Overall, our results corroborate crucial effects of these genes on the severity of EPEC infections.

To investigate the mechanisms utilized by EPEC to reduce the intestinal cell migration, a T3SS-deficient (Δ*escF*) EPEC strain was used. Initially, EPEC E2348/69 and T3SS-complemented strains inhibited cell migration; conversely, Δ*escF* EPEC did not reduce migration significantly. Therefore, we inferred that the presence of T3SS might have a role in reducing cell migration. In that regard, T3SS has a fundamental role in EPEC infection, as this system provides a portal whereby many effector proteins that affect the organization of the cell cytoskeleton are injected into host cells (32). Disassembly of cytoskeleton organization might compromise cell migration. In addition, in other *in vitro* and *in vivo* studies, deletions of various T3SS effectors of A/E pathogens were implicated in reducing disease severity that interferes with the formation of A/E lesions and avoiding epithelial barrier damage (33,34). Furthermore, other non-T3SS-dependent virulence-associated proteins were described in A/E pathogens (35). Some studies demonstrated that additional putative virulence factors, non-T3SS-dependent, such as pili and adhesins, were also necessary to achieve colonization in animal or cellular models (36,37).

The Rho GTPase proteins have a critical role in cytoskeletal rearrangement, and its activity is altered by EPEC effector proteins (38). Therefore, we determined whether EPEC strains altered expression of RhoA, Rac1, and Cdc42. In the present study, only levels of *Rac1* transcription were significantly decreased by EPEC strain E2348/69 and LDI001 at 3 h after infection, although levels of Rac 1 protein were not reduced by EPEC infection. However, Rac1 was active in all EPEC strains, except the T3SS-deficient strain. Therefore, we inferred that Rac-1 activation could have a role in the impairment of intestinal cell migration. In a previous study (38), Rac1 activating effectors, EspT and SopE, decreased cell detachment. As cell detachment is one of the steps of cell migration (10), we speculated that Rac1 activation delayed cell detachment and thereby reduced cell movement. Depending on the cell type and stimulus, active Rac1 could either promote or suppress cell migration. For example, melatonin reduces migration of endothelial cells through inhibition of Rac1 activation (39 [Bibr B40]), whereas infection of gastric epithelial cells with EPEC reduces cell migration associated with Rac1 inhibition (21). Based on the present study, we inferred that the decline of intestinal epithelial cell migration infected by EPEC occurred via activation of Rac1.

In conclusion, we inferred that EPEC strains altered intestinal epithelial cell migration *in vitro* via more than one mechanism and that this phenomenon may or may not be associated with cell death. Differences among wild-type strains in virulence genes profiles may account for different effects, with potential for *espL* and *nleE* genes having significant roles in impairment of cell migration. However, further studies are necessary to confirm the involvement of these virulence genes in intestinal cell migration. Moreover, the EPEC type III secretion system may be involved in inhibition of intestinal epithelial cell migration by EPEC 2348/69, as its deletion prevented the effect, which occurs through activation of Rac1. These data contributed to emerging basic and applied knowledge regarding pathobiology of EPEC infection and its association with the restoration of intestinal barrier functions after epithelial damage.

## Supplementary Material

Click here to view [pdf]
